# The Role of Circulating Tumor DNA in Ovarian Cancer

**DOI:** 10.3390/cancers16183117

**Published:** 2024-09-10

**Authors:** Anna Golara, Mateusz Kozłowski, Aneta Cymbaluk-Płoska

**Affiliations:** Department of Reconstructive Surgery and Gynecological Oncology, Pomeranian Medical University in Szczecin, Al. Powstańców Wielkopolskich 72, 70-111 Szczecin, Poland; aneta.cymbaluk@gmail.com

**Keywords:** ctDNA, ovarian cancer, chromosomal instability, somatic mutations, methylation

## Abstract

**Simple Summary:**

The diagnosis and treatment of ovarian cancer still pose many problems; so, it is important to search for effective biomarkers that will allow for the detection of changes in the early stages of the disease. There is more and more talk about the use of circulating tumor DNA in oncology as a promising biomarker and an aid in tailoring individual therapy to patients. We describe what changes we can observe in ctDNA in patients with ovarian cancer and how we can use this information in diagnosis and therapy.

**Abstract:**

Ovarian cancer is the deadliest of all gynecological diseases because its diagnosis and treatment still pose many problems. Surgical excision, hormone therapy, radiation, chemotherapy, or targeted therapy for eradicating the main tumor and halting the spread of metastases are among the treatment options available to individuals with ovarian cancer, depending on the disease’s stage. Tumor DNA that circulates in a patient’s bodily fluids has been studied recently as a possible novel biomarker for a number of cancers, as well as a means of quantifying tumor size and evaluating the efficacy of cancer therapy. The most significant alterations that we could find in the ctDNA of ovarian cancer patients—such as chromosomal instability, somatic mutations, and methylation—are discussed in this review. Additionally, we talk about the utility of ctDNA in diagnosis, prognosis, and therapy response prediction for these patients.

## 1. Introduction

**Figure 1:**
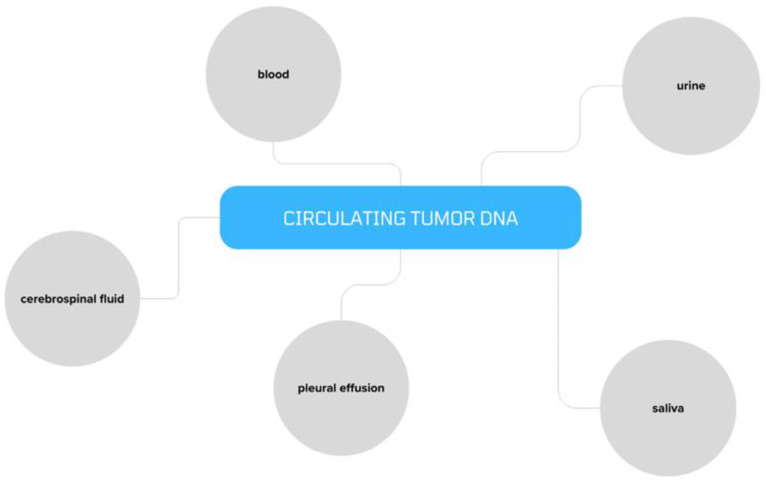
Body fluids that may contain ctDNA.

Ovarian cancer continues to be the most lethal of all gynecological diseases, despite advances in medicine and a plethora of research on the subject. Highly aggressive epithelial ovarian cancer, which is diverse in both its histology and molecular makeup, is the most often diagnosed kind of ovarian cancer [1]. When transperitoneal, hematopoietic, and lymphatic dissemination have already taken place, the great majority of ovarian malignancies are detected at an advanced stage (III/IV) [2]. Treatment options for patients with ovarian cancer vary depending on the stage of the disease and may include surgery, hormone therapy, radiation, chemotherapy, or targeted therapy to shrink and remove the primary tumor and stop the spread of any metastases [3]. Ovarian cancer metastases primarily occur through two pathways, each with distinct molecular mechanisms: transcoelomic metastasis, which involves the passive dispersion of tumor spheroids in peritoneal fluid and ascites, and hematogenous metastasis, which involves the spread of tumor cells in the bloodstream and their preferential seeding into the omentum through circulating tumor cells (CTCs) [4,5]. Cancer patients’ peripheral blood contains significant quantities of cell-free DNA (cfDNA), which can be utilized to identify certain molecular alterations linked to the growth of cancer [6]. It comprises all molecular alterations, including mutations specific to tumors such microsatellite instability (MI), loss of heterozygosity (LOH) [7], and DNA methylation [8]. The portion of free cellular DNA that is detected in circulation and has somatic mutations specific to tumors is known as circulating tumor DNA (ctDNA) [9]. DNA fragments known as ctDNA are removed from cancer tissues by apoptosis, necrosis, lysis, and active secretion into circulating body fluids, among others [10]. ctDNA can be detected in the blood, urine, saliva, pleural effusion, and cerebrospinal fluid [11] (Figure 1).

The state and size of the tumor, as well as lymphatic circulation, clearance, degradation, and other physiological blood processes, all affect the amount of ctDNA in bodily fluids [12,13]. It is possible to identify and measure ctDNA by sequencing, BEAMing technologies, and PCR. Even in the earliest stages of the disease, we find point mutations, copy number alterations, deletions, and epigenetic modifications in cancer tissues that are also present in ctDNA. Furthermore, it is reported that the mutational profiles of matched ctDNAs and solid tumors are concordant, indicating the possibility of using ctDNA for non-invasive ovarian cancer detection [14]. Since ctDNA has a brief half-life—roughly one hour—it is possible to track the advancement of tumors in real time. A non-invasive technique for identifying and tracking cancer, particularly in tumors that are challenging to biopsy, is serum ctDNA analysis, sometimes known as “liquid biopsy” [15,16].

## 2. ctDNA in Ovarian Cancer

Circulating mutation-specific cell-free tumor DNA (cftDNA) and circulating cell-free DNA (cfDNA) have been the subject of evaluation in recent years as potential novel biomarkers for various malignancies, as well as a means of measuring tumor volume and evaluating how well cancer treatment is working [17,18,19] (Table 1).

**Figure 2:**
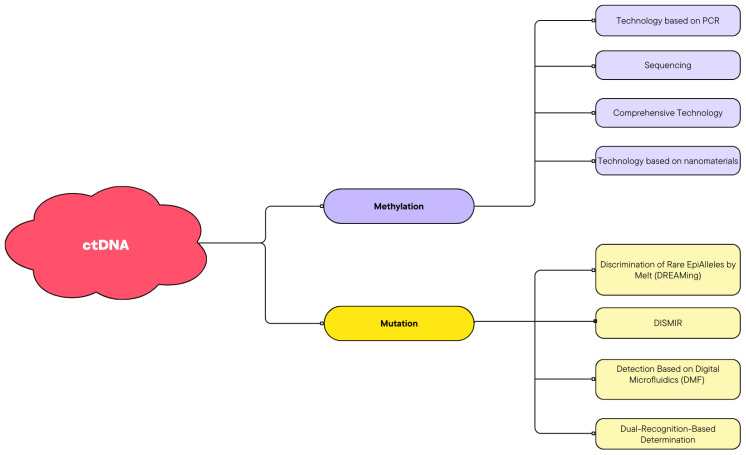
Detection technologies of ctDNA.

By applying the PCR technique, ctDNA may be quantified in plasma samples from patients with ovarian cancer and patients with borderline ovarian cancer [27]. In ovarian malignancies, ctDNA may represent increased tumor growth, metastasis, responsiveness to treatment, and recurrence [28]. We may determine the evolutionary paths of ovarian cancer by tracking the temporal heterogeneity of ctDNA, even at resolutions lower than exome scale [29]. The uterine cavity can be lavaged in order to identify ctDNA. Sixty-five patients with type II ovarian cancer (OC), five with endometrial cancer (EC), three with other malignancies, and twenty-seven with benign lesions affecting the gynecological organs had their samples taken by Maritschnegg et al. They used massively parallel sequencing and, in a subset, a singleton analysis to check for somatic mutations. Out of 30 individuals, 24 (80%) had specific mutations, 5 out of 5 had endometrial cancer, and 8 out of 27 (27%) had benign lesions. Because ovarian cancer cells are shed, they can be harvested through the lavage of the uterine cavity [30]. Possible methods of ctDNA detection are presented in Figure 2.

### 2.1. Chromosomal Instability

Ovarian cancer is characterized by chromosomal instability, and copy number alteration (CNA) profiling in cell-free DNA could be a useful diagnostic for identifying cancer in individuals with adnexal masses [31]. Quantitative PCR (qPCR) can be used to identify chromosomal rearrangements in the tumor and subsequently detect them in the plasma of patients. They exhibit a high specificity for cancer. Using cfDNA, a panel of customized tumor DNA-derived connections could be a useful tool for tracking cancer patients’ responses to treatment and likelihood of recurrence [32].

In a prospective study, Vanderstichele et al. examined low-coverage whole-genome sequencing of plasma from 68 patients who had adnexal tumors: 11 patients had benign carcinoma, and 57 patients had aggressive or borderline cancer. Patients with ovarian cancer had genome-wide z-scores in quantitative measures of chromosomal instability that were considerably greater than those in healthy controls or patients with benign diseases. Compared to serum CA-125 (Area Under the Curve (AUC) 0.78), or the risk of malignancy index (RMI, AUC 0.81), this study demonstrated superior malignancy identification (AUC 0.89). In individuals with high-grade serous ovarian cancer (HGSOC), AUC values in cell-free DNA assays climbed even further (AUC 0.94). This test had a 99.6% specificity, but cell-free DNA testing had a sensitivity that was two to five times higher than that of the CA-125 and RMI tests. In patients with adnexal masses, this approach improves the specificity of preoperative malignancy prediction by employing chromosomal instability as a readout [33].

In order to determine a genome-wide copy number instability score (CNI-Score) from low-coverage sequencing data, Braicu et al. used a clinical-grade technology to analyze cfDNA in blood samples from 109 individuals with high-grade epithelial ovarian cancer (EOC). The initial and repeated blood samples showed noticeably higher CNI-Scores. Following initial debulking surgery, a noteworthy decrease in CNI-Score was noted. The CNI-Scores of patients with primary EOC showed a 91% sensitivity and a 95% specificity when compared to controls. For high-grade EOC, quantification of ctDNA based on genomic instability as assessed by CNI-Score may be a biomarker with excellent diagnostic accuracy [34].

The methylation status and gene expression of frequently amplified HGSOC cancer genes are substantially correlated with somatic copy number changes (SCNAs). Multiregional HGSOC data allow for the differentiation of five dominant clonal driver SCNAs (chromosomal amplifications involving MYC, PIK3CA, CCNE1, KRAS, and TERT). The number of copies of the MYC chromosome is linked to the in vitro and clinical responses to paclitaxel as well as the in vitro response to mTORC1/2 inhibition. In the context of MYC-amplified HGSOC, activation of the mTOR survival pathway is statistically correlated with a higher frequency of SCNAs in PI3K pathway genes. Therefore, therapy can be modified in accordance with the identification of the co-occurrence of SCNA genes that are responsible for cloning [35].

### 2.2. Somatic Mutations

Over 96% of individuals with HGSOC have TP53 gene changes, which are recognized to be among the most prevalent mutations in cancer [36,37,38]. When DNA damage happens in normal cells, p53 keeps the cell cycle at the G1/S regulatory checkpoint, which results in growth arrest. Furthermore, in cases when DNA damage is irreparable, it triggers apoptosis and activates DNA repair proteins. Genes involved in the TP53 signaling pathway are impacted by neoadjuvant chemotherapy (NACT), a cancer treatment [39]. These mutations can serve as tailored indicators to track the amount of tumor growth and early alterations that can be utilized to forecast response and time to progression (TTP) [40]. In women with HGSOC, there is a baseline correlation between ctDNA and illness volume. Nonetheless, a shorter TTP is linked to a ≤60% drop in TP53MAF following a single treatment cycle [41]. In HGSOC, TP53mutctDNA exhibits promise as an ovarian cancer-specific biomarker to track therapy response [42]. TP53mut ctDNA levels had superior prognostic value than CA 125 after three months of treatment [43].

Otsuka et al. investigated whether DNA extracted from tumor tissue and plasma from 27 ovarian cancer patients had p53 mutations (exons 5–8). Tumor tissue from 12 patients (44%) contained p53 alterations. The same alterations were found in the plasma DNA of two of the twelve cases (16.7%) prior to surgery, but the altered DNA in the plasma was no longer traceable following the procedure. One patient had a p53 mutation when it resurfaced 16 months after surgery, and two months later, the patient passed away. This study showed that certain ovarian cancer patients, particularly those with more advanced stages of the disease, have tumor-derived DNA in their plasma [44]. According to Swisher et al., free tumor DNA was detectable in peritoneal fluid, circulating tumor DNA was present in one-third of women with ovarian cancer, and circulating tumor was an independent predictor of lower survival in multivariate analyses (*p* = 0.02). Of the 137 cancers examined, 69 patients (about 50%) had p53 mutations. Out of 30 cases, tumor DNA was found in the peritoneal fluid of 28 patients (93%) [45]. Additionally, Dobrzycka et al. evaluated the significance of KRAS gene mutations, blood plasma p53 antibodies (p53-Ab), and circulating free DNA (CFDNA) in the prognosis of 126 patients with epithelial ovarian cancer. Patients with high-grade serous tumors had higher frequencies of cfDNA and p53-Ab detection (*p* < 0.001). The overall survival rate was considerably worse for patients with serous, cfDNA- and p53-positive tumors than for those with negative tumors [46].

### 2.3. Methylation

A tiny volume of blood plasma (3 mL of blood) can be used to identify hypermethylation patterns in cell-free circulating tumor DNA, which is a straightforward and less invasive approach [47]. Tumor suppressor gene expression is changed by increased DNA methylation of promoter regions, an early event in the development of tumors [48,49,50]. Since DNA methylation is unique to cancer and may be seen in ctDNA, it may be used as a diagnostic to help identify OC early [51]. Certain benign and malignant ovarian cancers can also be distinguished from one another because of differential methylation of cell-free plasma DNA (cfpDNA) promoters [52]. In individuals with malignancies restricted to the ovaries (stage IA or B), promoter hypermethylation is frequently observed and very early in the ovarian carcinogenesis process. It is also detectable in the peritoneal fluid of patients with negative cytology [53].

Because human telomerase reverse transcriptase (hTERT) is essential for maintaining telomeres, it is linked to the development of OC. In addition to plasma samples from healthy women, Li et al. looked studied variations in the hTERT promoter methylation rates in tumor tissues and plasma samples from patients with benign ovarian tumors and patients with ovarian malignancies. The purpose of this study was also to determine whether patients with ovarian magnificent tumors had higher rates of hTERT promoter methylation and circulating tumor DNAs (ctDNA). Methylation-specific PCR (MSP) was used to evaluate the methylation of the promoter. In patients with ovarian magnificent tumors, this study found correlations between hTERT methylation and ctDNA (*p* = 0.001) and tumor tissue samples (*p* = 0.012) [54].

In order to confirm the assay’s specificity and sensitivity in identifying early-stage ovarian cancer (EOC), Zhang et al. tested a multiplex-methylation-specific PCR (MSP) assay employing cell-free DNA in blood. To build the multiplex-MSP assay, they chose seven potential genes with high methylation frequencies (APC, RASSF1A, CDH1, RUNX3, TFPI2, SFRP5, and OPCML). The preoperative serum methylation status of cell-free DNA was assessed in 202 patients, comprising 87 patients with benign ovarian tumors, 53 patients with ovarian cancers, and 62 healthy controls. In stage I EOC, the multiplex-MSP test demonstrated a sensitivity of 85.3% and a specificity of 90.5% [55]. More research is required, but the MSP test can be employed as a diagnostic tool for ovarian cancer in clinical oncology. To find the most precise OC methylation patterns, Widschwendter et al. examined 699 tumor and non-tumor tissues using a panel of three methylated genes. A specificity of 88.1% was found in a panel of 57.9% of women who had ovarian cancer within two years [56]. Additionally, fcDNA and an opioid binding protein/cell adhesion molecule (OPCML) can be used to diagnose people with early-stage EOC. Early-stage EOC patients have significantly changed OPCML methylation when compared to healthy donors (*p* < 0.0001), and distinct fcDNA methylation can be used to differentiate them from healthy donors [57].

The pathophysiology of EOC may be influenced by aberrant promoter methylation, which leads to the epigenetic inactivation of RASSF2A. As a result, RASSF2A gene methylation in plasma may serve as a molecular marker for the early identification of EOC [58]. Giannopoulou et al. investigated the methylation status of the RASSF1A promoter in primary tumors, tumor cell-free tissues anatomically close to the tumor, and matched circulating tumor DNA (ctDNA) samples from patients with high-grade serous ovarian cancers (HGSCs). The research employed methylation-sensitive high-resolution melting analysis (MS-HRMA) and real-time methylation-specific PCR (real-time MSP). HMSC primary tumor FFPE samples were split into two groups (group A, *n* = 67, and group B, *n* = 61). Group B also included corresponding plasma samples (*n* = 59) and morphologically matched neighboring tumor cell-free tissues (*n* = 58). Both groups’ primary tumors showed high levels of methylation at the RASSF1A promoter, but the morphologically adjacent tumor cell-free tissues showed lower levels of methylation. With real-time MSP, ctDNA can also be used to detect RASSF1A promoter methylation. On the other hand, when MS-HRMA was utilized with original tumor samples, there was a significant correlation between OS and RASSF1A promoter methylation (*p* = 0.023). Patients with HGSC have methylation of the RASSF1A promoter in the surrounding tissue of their tumor, which is significant prognostic information [59]. Patients with HGSC plasma ctDNA also have ESR1 methylation. ESR1 methylation in primary tumors and matched ctDNA are statistically significantly concordant. Better clinical outcomes are correlated with ESR1 methylation in patients with HGSC [60]. Higher values of the CDH1 gene are found in the plasma of patients, which makes it a real option in the non-invasive diagnosis of ovarian cancer [61].

## 3. Diagnostics

The best strategy for lowering the morbidity and death rate from cancer in humans is early detection and intervention. Transvaginal ultrasonography and serum cancer antigen 125 (CA-125) are employed as early screening techniques, yet they are inadequate since they do not detect OC early enough with both sensitivity and specificity [62,63]. Long-term research has indicated that ctDNA may be useful for the diagnosis of a wide range of malignancies [64].

Additionally, circulating cell-free mitochondrial and nuclear DNA may serve as biomarkers for epithelial ovarian cancer. After analyzing 104 patients, it was discovered that those with epithelial ovarian cancer had much higher plasma concentrations of circulating cell-free nuclear DNA and circulating cell-free mitochondrial DNA when compared to two other groups: one with benign ovarian diseases and the other with a healthy control group. Nevertheless, in patients with epithelial ovarian cancer, there was no correlation observed between the measurement of CA 125 and pathological characteristics or the amount of circulating cell-free DNA [65]. Preoperative plasma levels of total free DNA are significantly elevated in patients with invasive epithelial ovarian cancer (EOC), and elevated levels of plasma cell-free DNA are an independent predictor of death from ovarian cancer disease, according to Kamat et al.’s analysis of DNA extracted from the plasma of 164 women with EOC, 49 women with benign ovarian tumors, and 75 age-matched control women [66].

The characteristics of ovarian clear cell carcinoma (OCCC) include resistance to treatment, a dismal prognosis, and a connection to endometriosis. Using droplet digital PCR (ddPCR), Morikawa et al. found mutations in PIK3CA-H1047R and KRAS-G12D in cfDNA from OCCC patients. These mutations can be utilized to diagnose OCCC and predict its recurrence. It is also possible to increase the sensitivity of the ddPCR method and detect cfDNA containing PIK3CA-H1047R by cleaving wild-type PIK3CA using the CRISPR/Cas9 system [67].

Blood circulating cell-free DNA (ccfDNA) has a strong correlation with the development, amelioration, and recurrence of some malignant cancers [68]. Diagnostic indicators for ovarian cancer include the circulating ALU-219 concentration and the ALU-219/ALU-115 ratio, which is a measure of ccfDNA integrity. Monitoring plasma ALU levels, either by itself or in conjunction with other tumor markers, can be utilized for the prognosis and supplementary diagnosis of ovarian cancer [69].

There is a new FDA-approved FoundationOne^®^CDx (F1CDx) test on the medical market. It is an in vitro diagnostic device based on next-generation sequencing that can detect substitutions, insertion and deletion changes (indels), and copy number changes (CNA) in 324 genes and select gene rearrangements as well as genomic signatures including microsatellite instability (MSI) and cancer mutational load (TMB) using DNA isolated from formalin-fixed paraffin-embedded (FFPE) tumor tissue specimens. The test serves a diagnostic function in identifying patients who may benefit from treatment with targeted therapies. In ovarian cancer, the test detects BRCA1/2 alterations. The test also enables the detection of loss of heterozygosity of the genome (LOH) in formalin-fixed and paraffin-embedded (FFPE) ovarian cancer tissues. A positive homologous recombination deficiency (HRD) status in ovarian cancer patients is associated with improved progression-free status survival (PFS) after rucapari maintenance therapy [70]. There is also the CancerSEEK test, which uses a combination of 39 protein markers and amplicon-based cfDNA sequencing to detect a total of 16 major cancer types. In ovarian cancer, CancerSEEK achieves a sensitivity of 98% with a specificity of 99%. The test seems very promising, but its performance still requires independent validation and is still in the development phase and is currently not available to patients [71].

## 4. Prognosis and Prediction of Response to Treatment

Human epididymal protein 4 (HE4) [72] and CA125 (cancer antigen 125) [73] levels are measured in order to predict how well patients will respond to chemotherapy. Nevertheless, due to variations in epidemiological and clinical features, their precision and efficacy in forecasting chemo response fluctuate amongst patients [74]. Liquid biopsy, on the other hand, allows us to look at ctDNA and is used to measure minimal residual illness, evaluate drug resistance, and track therapy response [75]. The discovery of particular target gene mutations aids in the decision-making process when it comes to the appropriateness of treatment and the sophisticated detection of secondary resistance, with the ultimate goal of achieving early disease progression diagnosis [76,77]. For patients with advanced ovarian cancer, the preoperative serum RAB25 cfDNA level may be a helpful biomarker for predicting survival outcomes [78].

Treatment options for ovarian cancer can be personalized thanks to circulating tumor DNA (ctDNA). Using an NGS panel comprising nine genes—TP53, BRCA1, BRCA2, ARID1A, CCNE1, KRAS, MYC, PIK3CA, and PTEN—the usefulness of serial ctDNA testing was evaluated. A total of 201 women with ovarian cancer and 95 with mild or borderline disease were among 296 patients who underwent examination. A total of 811 blood samples were taken at baseline (first diagnosis or surgery) and then every three months after that. A total of 69.2% (139/201) of patients with ovarian cancer had somatic mutations at baseline; patients with benign or borderline illness did not have these mutations. At baseline and/or six months later, ctDNA detection was predicted by progression-free survival (PFS). When comparing patients whose ctDNA became undetectable at baseline and undetectable during follow up, PFS was significantly poorer in those with identifiable pathogenic mutations at baseline that continued during follow up. Between patients whose ctDNA result changed from positive to negative and those without pathogenic ctDNA mutations in baseline or follow-up samples, no differences were found. Compared to traditional radiological assessment or CA125 surveillance, ctDNA can identify disease recurrence earlier [79]. It is possible to find ctDNA with non-invasive prenatal testing (NIPT). When used in NIPT, low-coverage whole-genome sequencing analysis can detect copy number variation (CNV) in the ctDNA of patients with ovarian cancer both before and following chemotherapy. Patients who are resistant to or responsive to NAC have various CNVs, which can be exploited to treat cancer patients [80].

Somatic reversion mutations or intragenic deletions that restore BRCA1 or BRCA2 function result in resistance to PARP inhibition or platinum-based chemotherapy in carriers of germline BRCA1 or BRCA2 mutations [81]. cfDNA sequencing analysis can identify putative BRCA1/2 reversion mutations in patients with ovarian and breast cancer [82], which may help choose which patients to treat with PARP inhibitory medication [83,84]. Additionally, cell-free DNA exhibits independent prognostic value in bevacizumab-treated patients [85].

According to a number of studies, ctDNA testing can identify postoperative residual illness in EOC patients with good sensitivity and specificity [86]. The potential of serially obtained ctDNA to detect disease recurrence following primary tumor excision was evaluated in a prospective study involving 23 patients with ovarian cancer. According to this study, ctDNA had a 91% sensitivity in predicting tumor recurrence [87]. Research suggests that detectable levels of ctDNA in plasma can predict disease recurrence an average of seven months before a CT scan [88]. Vitale et al. used cfDNA sequences obtained from serum following primary debulking surgery to investigate TP53 mutations in 20 patients with high-grade serous ovarian cancer. They found that a greater percentage of patients with suboptimal debulking (67%) had detectable ctDNA (cfDNA with TP53 mutations) in comparison to patients with optimal volume reduction (45%) [89]. In women with ovarian cancer, ctDNA in postoperative serum samples may be a predictor of recurrence and the existence of microscopic residual disease [90,91].

The analysis of ctDNA derived from bodily fluids other than blood offers special benefits, including the ability to perform completely non-invasive serial sampling and more in-depth analysis of particular cancer types at particular anatomical locations. These benefits extend from exploratory research to routine clinical use [92].

## 5. Conclusions

1. Early diagnosis of ovarian cancer and selection of therapy tailored to the patient may be possible by detecting ctDNA in body fluids before treatment, but further research is needed.

2. Frequent ctDNA monitoring during cancer treatment enables treatment adjustment by shedding light on drug resistance brought on by genetic alterations.

3. Using ctDNA to track any remaining illness can help stop tumor recurrence.

## Figures and Tables

**Table 1:** Application of ctDNA in cancers.

Type of Cancer	Application.	DOI
Endometrial cancer	ctDNA can be used as a tool for risk stratification and disease monitoring.	[20]
Cervical cancer	ctDNA may be used as a complementary diagnostic tool rather than the sole decisive biomarker in cervical cancer.	[21]
	One helpful marker for predicting the return of cervical cancer is the detection of HPV ctDNA.	[22]
Breast cancer	ctDNA analysis in early breast cancer is helpful for screening, treatment evaluation, and detection of minimal residual disease (MRD).	[23]
Colorectal cancer	Postoperative ctDNA screening identifies patients at very high risk of relapse and offers proof of remaining illness.	[24]
Ovarian cancer	Elevated levels of cfDNA are observed in patients with ovarian cancer.	[25]
	When paired with additional molecular markers, cfDNA variables have the potential to serve as diagnostic biomarkers for ovarian cancer.	[26]
